# Altered Brain Functional Network in Parkinson Disease With Rapid Eye Movement Sleep Behavior Disorder

**DOI:** 10.3389/fneur.2020.563624

**Published:** 2020-10-27

**Authors:** Jiao Li, Qiaoling Zeng, Wen Zhou, Xiangwei Zhai, Chao Lai, Junlan Zhu, Shuwen Dong, Zhijian Lin, Guanxun Cheng

**Affiliations:** ^1^Department of Medical Imaging, Peking University Shenzhen Hospital, Shenzhen, China; ^2^Department of Neurology, Peking University Shenzhen Hospital, Shenzhen, China

**Keywords:** Parkinson disease, REM sleep behavior disorder, resting-state functional magnetic resonance imaging, functional network, graph theory

## Abstract

**Background and Objective:** Parkinson disease (PD) with rapid eye movement (REM) sleep behavior disorder (PD-RBD) tend to be a distinct phenotype with more severe clinical characteristics and pathological lesion when compared with PD without RBD (PD-nRBD). However, the pathological mechanism underlying PD-RBD remains unclear. We aim to use the resting-state functional magnetic resonance imaging (rs-fMRI) to explore the mechanism of PD-RBD from the perspective of internal connectivity networks.

**Materials and Methods:** A total of 92 PD patients and 20 age and sex matched normal controls (NC) were included. All participants underwent rs-fMRI scan and clinical assessment. According to the RBD screening questionnaire (RBDSQ), PD patients were divided into two groups: PD with probable RBD (PD-pRBD) and PD without probable RBD (PD-npRBD). The whole brain was divided into 90 regions using automated anatomic labeling atlas. Functional network of each subject was constructed according to the correlation of rs-fMRI blood oxygenation level dependent signals in any two brain regions and network metrics were analyzed using graph theory approaches. Network properties among three groups were compared and correlation analysis was made using distinguishing network metrics and RBDSQ scores.

**Results:** We found both PD-pRBD and PD-npRBD patients existed small-world characteristics. PD-pRBD showed a wider range of nodal property changes in neocortex and limbic system than PD-npRBD patients when compared with NC. Besides, PD-pRBD showed significant enhanced nodal efficiency in the bilateral thalamus and betweenness centrality in the left insula, but, reduced betweenness centrality in the right dorsolateral superior frontal gyrus when compared with PD-npRBD. Moreover, nodal efficiency in the bilateral thalamus were positively correlated with RBDSQ scores.

**Conclusions:** Both NC and PD patients displayed small-world properties and indiscriminate global measure but PD-pRBD showed more extensive changes of nodal properties than PD-npRBD. The increased centrality role in the bilateral thalamus and the left insula, and disruption in the right dorsolateral superior frontal gyrus may play as a key role in underlying pathogenesis of PD-RBD.

## Introduction

Rapid eye movement sleep behavior disorder (RBD) is an abnormal state of sleep, characterized by lost muscle atonia and abnormal dream acting behavior. It is thought to have a strong correlation with α-synucleinopathies, especially Parkinson disease (PD) (1, 2). It can occur either before or after the onset of typical motor symptoms of PD (3). Compared to PD patients without RBD (PD-nRBD), PD patients with RBD (PD-RBD) are more likely to have psychiatric disease, more severe autonomic dysfunction, motor manifestation, and cognitive impairment (4, 5). This indicates that PD-RBD tends to be a special disease phenotype, which means more serious pathology of Lewy bodies and clinical characteristics. However, we still know very little about the pathogenesis of RBD in PD.

Magnetic resonance imaging (MRI) is a non-invasive and convenient technique, which is widely used to explore the pathogenesis of central nervous system diseases. Some researchers successively explored structure changes in PD-RBD patients using voxel-based morphometry, diffusion tensor imaging and structural correlation network methods. They found PD-RBD has decreased volume in more or less cortical and subcortical structures such as thalamus, hippocampus, and cingulate cortex (6–12). In addition to brain structure, a few researchers also used the resting-state functional MRI (rs-fMRI) to search changes of brain function in PD-RBD. Gallea found PD-RBD showed decreased functional connectivity between pedunculopontine nucleus and anterior cingulate cortex (13). And Li found PD-RBD had decreased amplitude of low-frequency fluctuations in primary motor cortex and premotor cortex (14). Nevertheless, recent studies tend to regard neurodegenerative process as network-based neurodegeneration rather than only based on isolated regions (15). We must not only focus on the dysfunction of key brain regions, but also consider the collective effects between various brain regions and systemic overall-level disorders.

Suitably, the fMRI-based graph theory analysis allows the brain to be studied as a complex network, and it can reflect dynamic interactions between different brain regions by describing and analyzing the local and global characteristics of a graph composed of nodes and edges (16). This technique has been used to detect abnormal communication in the brain. Suo found the configurations of brain functional network in PD were perturbed and correlated with the severity of the disease (17). And another study found idiopathic RBD (iRBD) patients had reduced centrality in left superior parietal lobule when compared with healthy controls (18). However, the mechanism of overall brain functional network regulation and property changes in PD-RBD patients is still unknown.

The purpose of this study is to explore the underlying mechanism of PD patients with probable RBD (PD-pRBD) from the perspective of functional network regulation. We propose a hypothesis that PD-pRBD patients have characteristic brain functional network features and topological changes. Therefore, we construct a brain functional network of PD-pRBD patients using graph theory analysis to describe the characteristics of the brain network and explore its relationship with RBD symptom in PD patients.

## Materials and Methods

### Participants and Clinical Evaluation

The rs-fMRI data of PD patients and normal controls were from the Parkinson's Progression Markers Initiative (PPMI) database (www.ppmi-info.org/data). Written informed consent was obtained from all participants, and all PPMI sites was approved by their respective ethics committee. PD patients and NC were both divided into two groups according to the RBD screening questionnaire (RBDSQ) scores. A score of ≥6 of RBDSQ was considered as probable RBD (pRBD) in the present study (19). NC with pRBD were excluded. For an independent subject, we usually selected the baseline data, unless poor image quality, the follow-up data were selected. Then, 68 PD-npRBD patients, 32 PD-pRBD patients, and 20 NC were initially contained. Participants' neuropsychological performance was measured across a variety of cognitive tests. Verbal memory was assessed by the Hopkins Verbal Learning Test (HVLT). Verbal fluency was assessed by the Semantic Fluency Test (SF). Processing speed was assessed by the Symbol Digit Modalities Test (SDMT). Visuospatial ability was assessed by the Benton Judgement of Line Orientation Test (BJLO). Executive function was assessed by the Letter-Number Sequencing Test (LNS). Motor function was evaluated using Movement Disorder Society Unified Parkinson's Disease Rating Scale part three (MDS-UPDRS-III) and Hoehn & Yahr (H&Y) stage.

### FMRI Data Acquisition and Preprocessing

All fMRI images were acquired on 3.0 T Siemens scanners (Erlangen, Germany) at different centers using the ep2d_RESTING_STATE sequence. The acquisition parameters were as follows: repetition time = 2,400 ms; echo time = 25 ms; flip angle = 80°; voxel size = 3.3 mm^3^; slice thickness = 3.3 mm; and each brain volume comprised 40 axial slices and one functional run contained 210 brain volumes. The fMRI images were preprocessed using DIPABI software (http://rfmri.org/dpabi). The steps were as follows: removal of the first 10 volumes, slice timing, realignment, spatial normalization through EPI templates, smoothing with a Gaussian kernel of 6 × 6 × 6 full width at half maximum, linear detrending, the regression of nuisance and temporal band-pass filtering (0.01–0.1 Hz). Finally, after ruling out poor-quality images, 62 PD-npRBD patients, 30 PD-pRBD patients, and 20 NC were retained to final analysis.

### Construction of Network

The network was constructed using GRETNA (http://www.nitrc.org/projects/gretna/). First, the whole brain was divided into 90 cortical and subcortical regions of interest by using the automated anatomic labeling atlas with each region representing a network node. Next, to define the edges of the network, we acquired the mean time series of each region and calculated the Pearson correlations of the mean time series between all pairs of nodes. This resulted in a 90 ^*^90 Pearson correlation matrix for each participant. The matrix was binary according to a predefined threshold, if the Pearson correlation coefficient between any two regions exceeds the threshold, there will be an edge between that two regions (8).

### Network Analysis

We applied a sparsity threshold (0.05–0.5, with an interval of 0.05) to all correlation matrices. For the brain network at each sparsity level, we calculated both global and nodal network properties and the area under the curve (AUC) for each property over the sparsity range. The global properties include small-world, network efficiency, assortativity, synchronization and hierarchy. The nodal properties include nodal clustering coefficient, nodal shortest path length, nodal efficiency, nodal local efficiency, nodal degree centrality and nodal betweenness centrality.

Small-world properties indicate that the information segmentation and integration achieve a balance maximizing the efficiency of information transfer with a relatively low wiring cost. Network efficiency measures the global efficiency of parallel information transfer in a network. Assortativity reflects the connection trend of nodes which have similar numbers of edges. Synchronization measures the possibility all nodes fluctuate in the same wave pattern. The hierarchy coefficient reflects the presence of a hierarchical organization of network (20).

The clustering coefficient of a node measures the possibility its neighbor interconnect. The nodal local efficiency measures the communication efficiency among its first neighbors when the node is removed. The shortest path length and efficiency of a given node quantifies the efficiency of parallel information transfer of that node in the network. The degree centrality and betweenness centrality of a given node reflects its importance on information transfer (20).

### Statistical Analysis

The analyses of demographic and clinical data and global network properties were performed with SPSS version 25.0. Continuous variables were compared use one-way analysis of variance (ANOVA) with pairwise *t*-test (with Bonferroni correction) or Kruskal-Wallis 1-way ANOVA with pairwise Mann -Whitney *U* Test (with Bonferroni correction) according to its distribution. Categorical variables were compared use Chi-square statistics. We choose *P* < 0.05(two-tailed) to indicate that the difference was statistically significant.

The analysis of the AUC of nodal network properties was performed using general linear model with age, gender, and head motion as nuisance covariates. For multiple comparisons of nodal network properties, we use false-positive correction [*P* = 1/90 (1/*N*) = 0.01, *N* means 90 compared nodes in total, which implies that the expected average false positive rate is <1 in each analysis] (21–24), and use Bonferroni correction to adjust the *p*-value when comparing the nodal network properties in the general linear model among three groups (PD-pRBD, PD-npRBD, and NC).

Finally, Spearman correlations were analyzed to examine relationships between networks properties with significant group effects and clinical characteristics with significant group effects (RBDSQ scores). We did correlation analysis cross the whole patient group (PD-pRBD and PD-npRBD).

## Results

### Demographic and Clinical Characteristic

Demographic and clinical data of 62 PD-npRBD patients, 30 PD-pRBD patients, and 20 NC were presented in Table 1. There was no significant difference except RBDSQ scores between PD-pRBD and PD-npRBD groups (*p* < 0.001). The education years of NC were higher than two PD patient groups (*p* < 0.001). The MDS-UPDRS-III score of NC were lower than PD groups (*p* < 0.001).

**Table 1:** Demographic and clinical characteristics of PD patients and controls.

**Variables**	**NC (*n* = 20)**	**PD-npRBD (*n* = 62)**	**PD-pRBD (*n* = 30)**	***P*****-value**			
				**NC vs. PD-npRBD vs. PD-pRBD**	**PD-npRBD vs. PD-pRBD**	**NC vs. PD-npRBD**	**NC vs. PD-pRBD**
Number (F/M)1^a^	4/16	24/38	7/23	0.16	–	–	–
Age, years^b^	64 ± 9.448	61.32 ± 10.390	61.87 ± 9.566	0.583	–	–	–
Education, years^c^	16 (14.5–17.75)	16 (13.75–17.25)	16 (14-18)	**<0.001**	0.374	**<0.001**	**<0.001**
BJLO score^c^	13 (11-15)	13 (12-14)	13 (12-14)	0.741	–	–	–
LNS score^c^	11 (10-12)	12 (10-13)	11 (7.75–12.25)	0.119	–	–	–
SF score^b^	53.20 ± 10.483	55.69 ± 10.167	49.33 ± 10.857	0.076	–	–	–
SDMT score^b^	47.833 ± 11.978	45.929 ± 8.719	42.083 ± 7.891	0.087	–	–	–
HVLT Total Recall score^b^	48.33 ± 9.225	48.77 ± 11.815	45.53 ± 16.205	0.523	–	–	–
HVLT Delayed Recall score^b^	45.93 ± 10.433	49.58 ± 12.430	46.67 ± 14.731	0.452	–	–	–
HVLT Retention score^c^	44 (39-50)	53.5 (43.75–56)	49.5 (40.25–56.25)	0.178	–	–	–
HVLT Discrimination Recognition score^c^	52 (49–59)	53 (44–57)	56 (45–57.25)	0.88	–	–	–
MDS-UPDRS-III score^c^	0 (0–1)	19 (12-25)	25 (14-36)	**<0.001**	0.081	**<0.001**	**<0.001**
Hoehn & Yahr (H&Y) stage^d^	–	2 (1-2)	2 (1.75–2)	–	0.292	–	–
RBDSQ score^c^	3 (2-4)	3 (2-4)	9 (7-11)	**<0.001**	**<0.001**	0.819	**<0.001**

*Data shown as mean ± standard deviation or median (quartiles 25–75%) when appropriate. Bold for P < 0.05*.

a *Chi-square test*.

b *ANOVA*.

c *Kruskal-Wallis 1-way ANOVA*.

d *Mann -Whitney U-test*1.

*When ANOVA showed significant differences, we performed post hoc comparisons using Students-Newman-Keuls. When Kruskal-Wallis 1-way ANOVA showed significant differences, we performed a pairwise comparisons*.

*PD-pRBD, Parkinson's disease with probable rapid eye movement sleep behavior disorders; PD-npRBD, Parkinson's disease without probable rapid eye movement sleep behavior disorders; BJLO, Benton Judgement of Line Orientation Test; LNS, Letter-Number Sequencing Test; SF, Semantic Fluency Test; SDMT, Symbol Digit Modalities Test; HVLT, Hopkins Verbal Learning Test; MDS-UPDRS, Movement Disorder Society Unified Parkinson's Disease Rating Scale; RBDSQ, RBD screening questionnaire; ANOVA, one-way analysis of variance*.

### Small-World Characteristics

**Figure 1:**
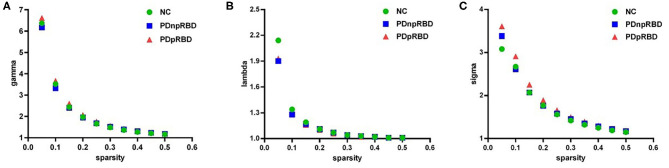
The small-world properties of brain functional network. **(A)** gamma (normalized cluster coefficient) is larger than 1, **(B)** lambda (normalized characteristic path length) is close to 1, **(C)** sigma (small-worldness) is larger than 1, showing that the brain functional networks in three groups have small-world characteristics. NC, normal controls; PDnpRBD, PD patients without probable rapid eye movement sleep behavior disorder; PDpRBD, PD patients with probable rapid eye movement sleep behavior disorder.

We found that over the sparsity value of 0.05–0.5, gamma was larger than 1, lambda was near 1 and sigma was larger than 1 for all functional connectivity networks of three groups (Figure 1). This indicated that typical small-world characteristic existed among NC, PD-npRBD, and PD-pRBD patients. There was no significant difference among three groups of small-world characteristics (aGamma, aLambda, aSigma) (*p* > 0.05).

### Global Network Measures

We did not find any significant difference of global network measures (small-world, network efficiency, assortativity, synchronization and hierarchy) through AUC analyses among the three groups.

### Regional Network Measures

#### Comparisons Between NC and PD-pRBD (PD-npRBD)

**Figure 2:**
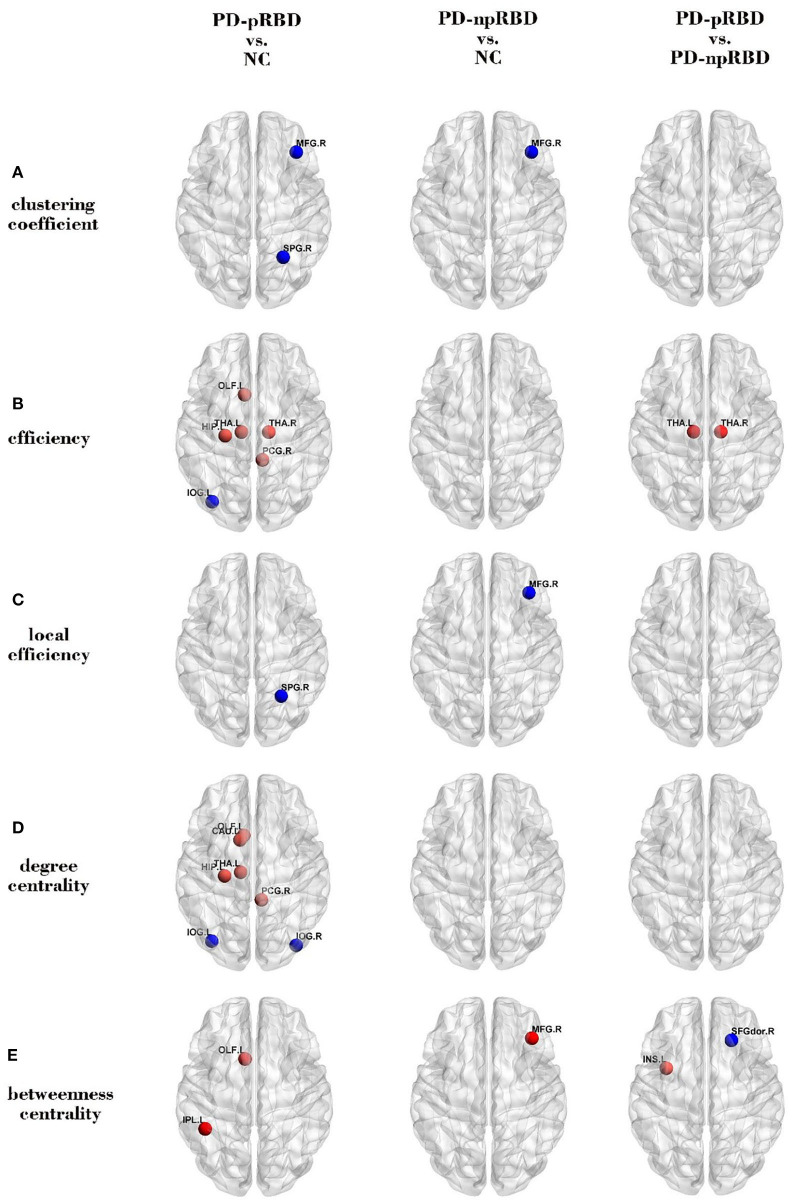
Comparisons of regional network measures. Group differences of **(A)** clustering coefficient, **(B)** efficiency, **(C)** local efficiency, **(D)** degree centrality, and **(E)** betweenness centrality between any two groups (PD-pRBD vs. NC, PD-npRBD vs. NC, and PD-pRBD vs. PD-npRBD).The red (blue) nodes in three panels, respectively, indicated increased (decreased) regional network measures in PD-pRBD (vs. NC), PD-npRBD (vs. NC), and PD-pRBD (vs. PD-npRBD). The results were visualized using the BrainNet Viewer (Beijing Normal University, http://www.nitrc.org/projects/bnv/). NC, normal controls; PD-npRBD, PD patients without probable rapid eye movement sleep behavior disorder; PD-pRBD, PD patients with probable rapid eye movement sleep behavior disorder.

When compared with NC, PD-pRBD had wider regions with increased nodal measures than PD-npRBD. PD-pRBD showed increased nodal measures in frontal lobe (left olfactory, *p* = 0.006), limbic lobe(right post cingulum, *p* = 0.005; left hippocampus, *p* = 0.003), and sub cortical gray nuclei (left and right thalamus, *p* = 0.004, *p* = 0.006) for nodal efficiency; in frontal lobe (left olfactory, *p* = 0.009), limbic lobe (right post cingulum, *p* = 0.005; left hippocampus, *p* = 0.003), and sub cortical gray nuclei (left caudate, *p* = 0.005; left thalamus, *p* = 0.009) for nodal degree centrality; in frontal lobe (left olfactory, *p* = 0.004) and parietal lobe (left inferior parietal gyrus, *p* = 0.008) for nodal betweenness centrality. PD-npRBD showed increased nodal measures in frontal lobe (right middle frontal gyrus, *p* = 0.004) for betweenness centrality when compared with NC (Figure 2).

Also, when compared with NC, PD-pRBD had wider regions with decreased nodal measures than PD-npRBD. PD-pRBD showed decreased nodal clustering coefficient in frontal lobe (right middle frontal gyrus, *p* = 0.007) and parietal lobe (right superior parietal gyrus, *p* = 0.003), decreased nodal efficiency in occipital lobe (left inferior occipital gyrus, *p* = 0.008), decreased nodal local efficiency in parietal lobe (right superior parietal gyrus, *p* = 0.006), decreased nodal degree centrality in occipital lobe (bilateral inferior occipital gyrus, *p* = 0.003, *p* = 0.006). PD-npRBD showed decreased nodal clustering coefficient and local efficiency in right middle frontal gyrus (*p* = 0.001, *p* = 0.006) (Figure 2).

#### Comparisons Between PD-npRBD and PD-pRBD

**Figure 3:**
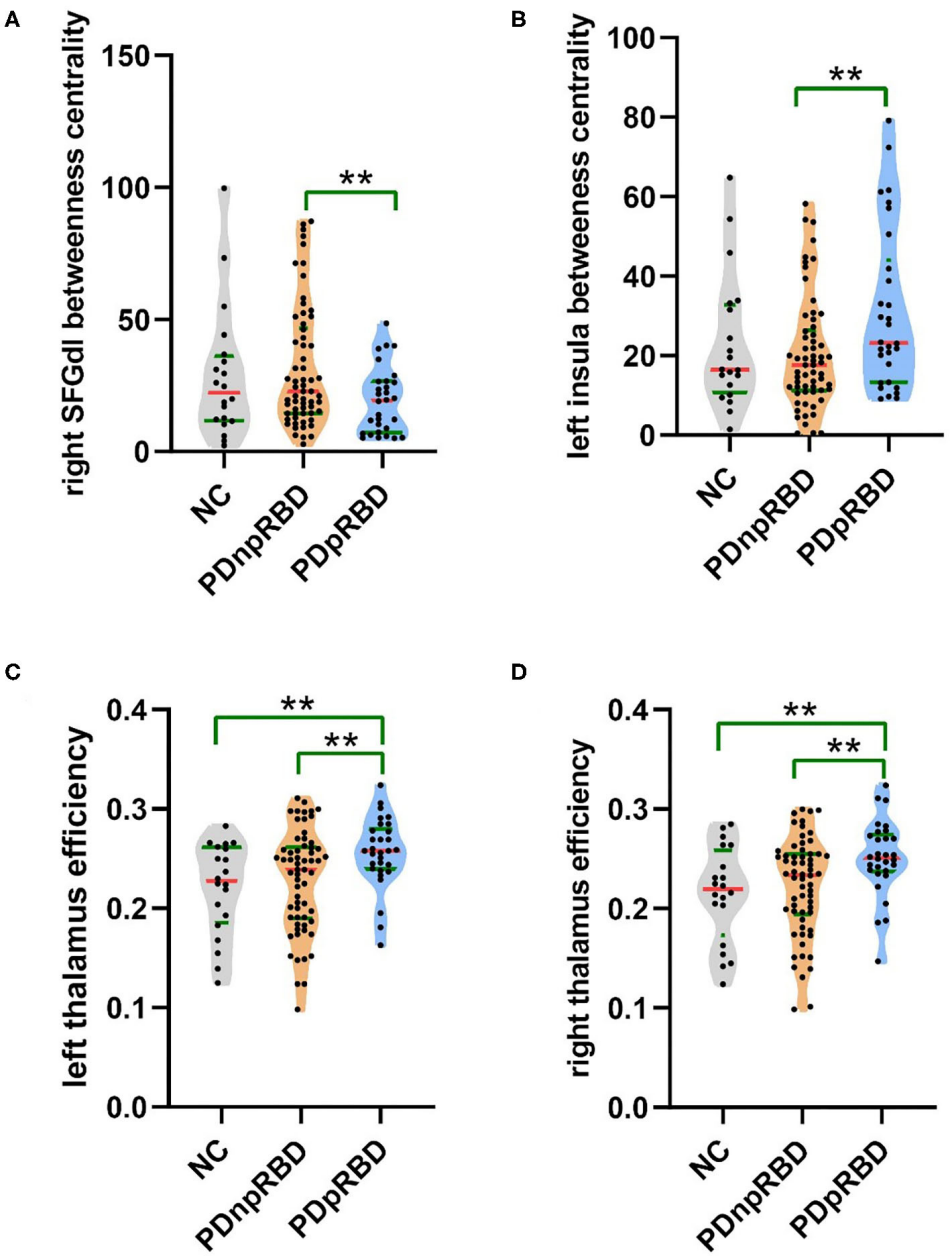
Violin plot of different regional network measures between two groups of PD. Descriptions of the betweenness centrality of the right SFGdl and the left insula, and efficiency of the bilateral thalamus among three groups. **(A)** The betweenness centrality of the right dorsolateral superior frontal gyrus was decreased, **(B)** the betweenness centrality of the left insula was increased, and **(C,D)** the efficiency of the bilateral thalamus was increased in PD-pRBD patients when compared with PD-npRBD patients. The **indicated that the difference was significant (*P* < 0.01). NC, normal controls; PDnpRBD, PD patients without probable rapid eye movement sleep behavior disorder; PDpRBD, PD patients with probable rapid eye movement sleep behavior disorder; SFGdl, dorsolateral superior frontal gyrus.

When comparing with PD-npRBD, PD-pRBD showed increased nodal efficiency in the bilateral thalamus (*p* = 0.007, *p* = 0.006) and increased betweenness centrality in the left insula (*p* = 0.006), as well as decreased betweenness centrality in the right dorsolateral superior frontal gyrus (*p* = 0.004) (Figures 2, 3).

### Correlation Analysis

**Figure 4:**
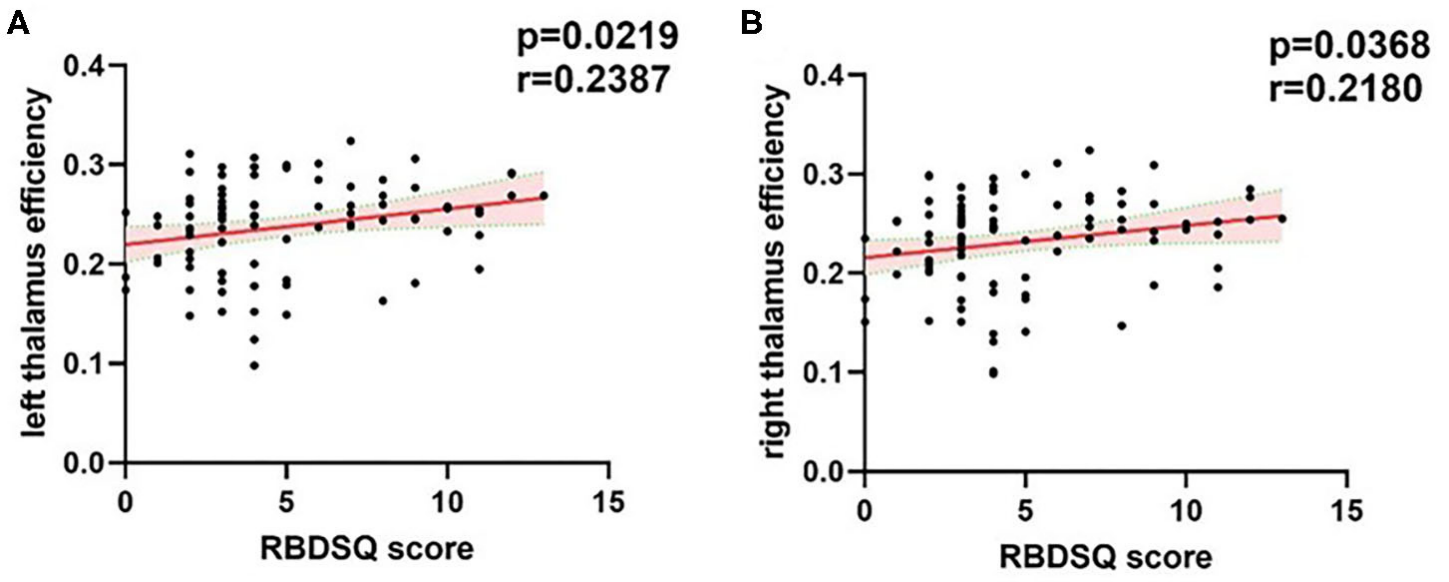
Correlations of the bilateral thalamus efficiency with RBDSQ scores. **(A)** The significant positive correlations between left thalamus efficiency and RBDSQ scores. **(B)** The significant positive correlations between right thalamus efficiency and RBDSQ scores. The red solid regression line indicated that the correlation was significant (*P* < 0.05). The pink shade represents the 95% confidence interval. The correlation analysis was applied cross the whole patient group. RBDSQ, rapid eye movement sleep behavior disorder screening questionnaire.

We found that the RBDSQ score correlated positively with nodal efficiency of bilateral thalamus (*r* = 0.2387, *p* = 0.0219; *r* = 0.218, *p* = 0.0368) (Figure 4).

## Discussion

In this study, we used graph theory analysis to explore the changes of topology properties of brain functional networks in PD-pRBD an PD-npRBD patients. There are two principal findings in the present study. Firstly, the functional networks of both PD groups (PD-pRBD and PD-npRBD) retained small-world properties and global function. Secondly, PD-pRBD had wider nodal parameter changes than PD-npRBD when compared with NC, the efficiency of the bilateral thalamus and the betweenness centrality of the left insula were increased and the betweenness centrality of the right dorsolateral superior frontal gyrus was decreased in PD-pRBD patients when compared with PD-npRBD patients. And the bilateral thalamus efficiency was positively correlated with RBDSQ scores.

### Small-World Properties and Global Measure of PD and NC

Small-world properties indicate that the information segmentation and integration achieve a balance maximizing the efficiency of information transfer with a relatively low wiring cost. Our study found PD patients and NC exhibited typical features of the small-world, and there was no significant difference in small-world properties and global measure among three groups. This was consistent with previous studies (11, 17, 25, 26). That is to say, the information of brain can be integrated effectively in both PD patients and NC. And the global network properties of PD patients and NC were similar. However, there were also some studies that showed different results from ours. For example, Suo found the clustering coefficient and global efficiency of PD patients was decreased, and the local efficiency and characteristic path length was increased when compared to NC (17). Luo found the PD group showed lower clustering coefficient and local efficiency than NC (26). The discrepancy of these results may be due to the difference of severity of PD patients, parameters of image acquisition, method of processing, and the heterogeneity of PD patients.

### Altered Nodal Measure in PD-pRBD Compared With NC

In this study, we found PD-pRBD patients showed extensive changes of nodal measure when compared with NC. The nodes whose network property were increased mainly located in left olfactory, left inferior parietal gyrus, right post cingulum, left hippocampus, bilateral thalamus, left caudate. And the nodes whose network property were decreased mainly located in right middle frontal gyrus, right superior parietal gyrus, bilateral inferior occipital gyrus. These nuclei were often mentioned in studies about PD or PD-RBD patients. A fMRI study comparing functional network of PD and NC reported the degree was decreased in occipital gyrus and increased in inferior parietal gyrus, post cingulum (27). Another similar study about PD patients also reported decreased node centralities and connectivity strength in occipital regions (26). Moreover, some PET studies about iRBD reported increased metabolism in thalamus and hippocampus, decreased metabolism in occipital lobe (28, 29). This may indicate that the decreased occipital function and increased function of limbic system and sub cortical gray nuclei play an important role in the development of PD and RBD. As for the entorhinal cortex, some studies reported that olfactory impairment was a marker of RBD and PD (30). Pathological studies about PD-RBD also reported accumulation of α-synuclein in entorhinal cortex (31). So, the increase of network property in left olfactory may be a kind of compensation.

### Decreased Betweenness Centrality of Right Dorsolateral Superior Frontal Gyrus (PD-pRBD vs. PD-npRBD)

Although both PD-pRBD and PD-npRBD patients kept the small-world properties and normal global communication function, we found PD-pRBD had a wider range of nodal properties changes than PD-npRBD patients, especially in the dorsolateral superior frontal gyrus (SFGdl). We found PD-pRBD had decreased betweenness centrality in the SFGdl when compared with PD-npRBD (*p* = 0.0036, *t* = −2.99). The SFGdl is located in the upper prefrontal cortex and forms the frontoparietal network. The frontoparietal network is considered to be a flexible cognitive control center, which regulates and integrates high-level emotions and consciousness (32). PD-pRBD patients have a characteristic decentralization of the SFGdl, which may weaken the frontoparietal network function and reduce the ability of emotional and cognition regulation. There were some studies presented that PD patients with RBD tend to perform worse in cognitive test (8, 10, 33). Therefore, some scholars believe that the characteristic decentralization of SFGdl in PD-pRBD patients may be related to the decline in cognitive regulation. However, our study did not find a significant decline in cognitive function in the PD-pRBD patients. This may be because the sample size was insufficient to cause significant changes in clinical cognition performance in our study. Of course, the functional impairments we observed in PD-pRBD patients may just reflects the nature of RBD itself associating with neurodegeneration rather than worse cognitive performance as reported in iRBD patients (34). The association of the SFGdl and RBD needs more studies with large sample size to validate.

### Increased Efficiency of Bilateral Thalamus (PD-pRBD vs. PD-npRBD)

Thalamus is a small structure within the brain located just above the midbrain between cerebral cortex and brain stem and has extensive nerve connections to both. The main function of the thalamus is to relay motor and sensory signals to the cerebral cortex. It also regulates sleep and wakefulness. Some previous morphometry studies showed markedly reduced gray matter volume in the bilateral thalamus of PD-pRBD patients in comparison with PD-npRBD patients and the volume of thalamus is negatively correlated with RBDSQ scores (7, 8, 12). And some previous functional studies also reported changes of thalamus function. Positron emission tomography (PET) studies have reported increased metabolism in thalamus of iRBD (35, 36). A fMRI study reported that resting-state thalamo-occipital functional connectivity was increased in iRBD patients (37). And a pathologic study reported that PD patients with sleep disorder showed more severe α-synuclein pathology in thalamus (31). Combined with our findings that efficiency of the bilateral thalamus was increased in PD-pRBD and correlated positively with RBDSQ scores, we speculate that increased thalamus efficiency is the compensation for the reduced thalamus volume and more severe pathology to maintain normal function of brain global information transfer. The number of studies exploring changes of PD-RBD functional connectivity was small. Future studies could focus on functional connectivity of thalamus and the combination of volume changes in PD-RBD patients.

### Increased Betweenness Centrality of Left Insula (PD-pRBD vs. PD-npRBD)

The cortical limbic system related to controlling emotions is considered to be involved in RBD, as the behaviors observed in RBD are violent and the recalled dreams are unhappy and fearful (38). The insular cortex is considered as limbic-related cortex. The insula is well-situated for the integration of information relating to bodily states into higher-order cognitive and emotional processes. It receives information from “homeostatic afferent” sensory pathways through the thalamus and sends output to some other limbic-related structures, such as the amygdala and ventral striatum (39). A study focused on structural correlation network reported that the nodal betweenness and degree of limbic system was increased (11). And a PET study of iRBD reported increased metabolism in hippocampus. These results suggest that the function of the limbic system was increased in RBD patients. This is consistent with our results. In addition, some previous studies found that compared with PD-npRBD, PD-pRBD patients showed reduced volume of the left insula (6). Other studies reported a decrease in the volume of other limbic systems such as the left posterior cingulate and hippocampal gray matter (10). And a pathologic study reported that PD patients with sleep disorder showed more severe α-synuclein pathology in limbic system than those without sleep disorder (31). Based on these results, we speculate that the increased betweenness centrality of the left insula is compensatory to volume decrease and pathological involvement to maintain the overall information conversion efficiency of the brain. However, some studies based on morphometry did not find any significant difference in limbic system (7, 12, 14). The difference may be due to the size of the sample and the severity of the patient's symptoms. In the future, it is necessary to study large samples and combine multi-modal images.

### Limitations

There are several limitations in this study. First, we used the RBDSQ score to group PD patients rather than polysomnography. However, the RBDSQ score used to identify RBD have a high sensitivity (96%) and specificity (85%) (40). Second, the cerebellum was not included as nodes because many images we downloaded did not coverage of the cerebellum completely. Future studies covering these regions are needed to further explore the topological structures for the brain networks of PD-RBD patients. Third, we carried out a cross-sectional study and the symptom of patients were relatively mild. In the future, longitudinal follow-up studies and patients with more severe symptoms should be included to help us further understand the pathogenies of PD-RBD.

## Conclusions

Both NC and PD patients displayed small-world properties and indiscriminate global measure but PD-pRBD showed more extensive changes of nodal properties than PD-npRBD. The increased centrality role in the bilateral thalamus and the left insula, and disruption in the right dorsolateral superior frontal gyrus may play as a key role in underlying pathogenesis of PD-RBD.

## Data Availability Statement

All datasets generated for this study are included in the article/supplementary material.

## Ethics Statement

The studies involving human participants were reviewed and approved by Parkinson's progression markers initiative ethics committee. The patients/participants provided their written informed consent to participate in this study.

## Author Contributions

JL and QZ designed the study, performed the experiments, and wrote original draft. WZ conducted project supervision and administration. XZ conducted statistical analysis. CL, JZ, and SD performed the experiments. GC and ZL reviewed and edited the manuscript. All authors contributed to the article and approved the submitted version.

## Conflict of Interest

The authors declare that the research was conducted in the absence of any commercial or financial relationships that could be construed as a potential conflict of interest.
